# A Review Of Effective Interventions To Improve Emotional Risk Factors Of Anxiety, Stress, Depression In Infertile And Infertile Patients Undergoing Treatment With Assisted Reproductive Techniques

**DOI:** 10.1192/j.eurpsy.2022.2254

**Published:** 2022-09-01

**Authors:** N. Salarian, Z. Hamzehgardeshi, Z. Shahhosseini

**Affiliations:** 1 Mazandaran University of Medical Sciences, Department Of Reproductive Health And Midwifery, sari, Iran; 2 Mazandaran University of Medical Sciences, Department Of Reproductive Health And Midwifery, sari, Iran

**Keywords:** emotional risk factors, infertile patients, Stress, Anxiety

## Abstract

**Introduction:**

Infertility is a major problem in life and affects the lives of infertile couples in every way

**Objectives:**

Infertility is associated with several negative reactions and emotional problems. Review of effective interventions for improving emotional risk factors In infertile and infertile patients undergoing treatment

**Methods:**

A comprehensive narrative review of the studies was conducted. Databases such as Web of Science, Science Direct, Cochrane Library, Scopus, PubMed/Medline, Clinical Key, and MAGIRAN were retrieved from May 10 to August 8, 2021, with no time limit. After reviewing the abstract and the full text of the articles, 32 articles were selected for writing. The methodological quality of the article was assessed based on the Cochrane Risk of Bias

**Results:**

Interventions were divided into two subgroups of mind-body, and web-based CBT. Mind-body interventions generally show the anxiety, stress and depression reduction and Possible improvement in pregnancy rate, but most of these programs require extensive financial resources.The results of web-based,showed that using online CBT approach can greatly reduce stress and anxiety,due to increased use of the internet, non-collaborative, cheap and private treatment of web-based interventions, this method can be used as a way along with other treatments to reduce these negative reactions.

**Conclusions:**

According to the present study CBT methods, application and Internet-based interventions can be used as appropriate counseling methods in reducing stress, anxiety and improving pregnancy outcomes in infertile patients. This information can be used as a proper source to select appropriate counseling methods for health care providers, midwives, and treatment staff involved in infertility patients.

**Disclosure:**

No significant relationships.

